# 631. Using a commercially available assay that measures cytomegalovirus (CMV)-specific CD4+ and CD8+ T-cell immunity to predict protection against CMV: Results from a prospective, blinded clinical trial

**DOI:** 10.1093/ofid/ofad500.697

**Published:** 2023-11-27

**Authors:** Nouf K Almaghlouth, Panos Arvanitis, Kendra Vieira, Dimitrios Farmakiotis

**Affiliations:** Mountain View Regional Medical Center, Las Cruces, New Mexico; The Warren Alpert Medical School of Brown University, Providence, Rhode Island; Divisions of Infectious Diseases,the Warren Alpert Medical School of Brown University, providence, Rhode Island; Division of Infectious Diseases, The Warren Alpert Medical School of Brown University, providence, Rhode Island

## Abstract

**Background:**

Cytomegalovirus (CMV) infection is associated with significant morbidity and mortality among kidney transplant recipients (KTR). Given toxicities and costs of antivirals, there is growing interest in assays that measure CMV-specific T-cell immunity (TCI), which may predict protection against clinical infection. The Viracor® CMV TCI Panel (TCIP) uses flow cytometry and intracellular cytokine staining to measure %CMV-specific CD4+ and CD8+ T-cells; it is the only commercially available TCI assay in the US. In this investigator-initiated study, we evaluated the performance of the TCIP in predicting clinically significant CMV events. To our knowledge, this is the first prospective blinded clinical study of the TCIP.

**Methods:**

We enrolled consecutive donor (D) or recipient (R) CMV seropositive KTR, who had TCIP testing monthly until either discontinuation of valganciclovir prophylaxis or the primary outcome. We also enrolled KTR with low-level untreated DNAemia, or after completion of valganciclovir treatment, to evaluate assay predictive values for progression or relapse of CMV infection, respectively. The primary outcome was CMV DNAemia (tested at the discretion of the treating provider for symptoms or laboratory findings), prompting treatment initiation. Secondary outcomes were any DNAemia and DNAemia >1000 IU/mL. Treating providers were unaware of TCIP results.

**Results:**

We included 46 KTR. Median age was 50.5 (range 18-74) years. 73.6% identified as men, 10.8% Black, 8.7% Hispanic; 41.3% had CMV D+/R- status. KTR with CMV events had significantly lower %CMV-specific CD8+ T-cells (Fig. 1A, *p*=0.024), and the CMV protection ROC AUC was significant (Fig. 1B: AUC 0.78, 95%CI 0.55-0.99, *p*=0.026). The positive predictive values of both CD4+ and CD8+ T-cell positivity >0.2% for CMV protection were 96.3% (95%CI 81-99.9%) for the primary outcome, 92.6% (95%CI 75.7-99.1%) for any DNAemia, and 100% (one-sided 95%CI 87.2%) for DNAemia >1000 IU/mL.

**Figure 1.**
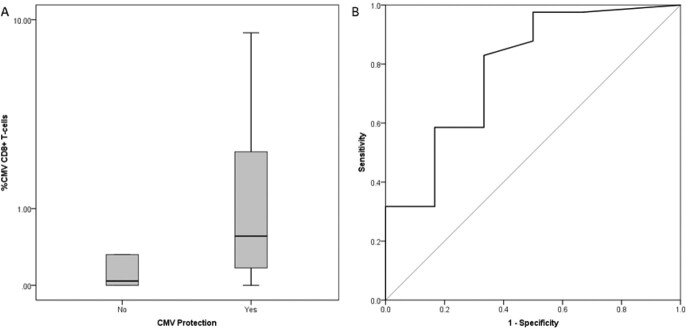
A. Comparison of CMV-specific %CD8+ T-cells between patients with or without (protected) CMV events. B Receiver Operating Characteristic (ROC) curve for CMV protection.

**Conclusion:**

The TCIP exhibited good performance in predicting protection from CMV events and could be a useful adjunct tool in clinical decision-making, regarding initiation or extension of CMV prophylaxis or treatment, as well as adjustments in immunosuppression.

**Disclosures:**

**Panos Arvanitis, MS**, NIH: Grant/Research Support|NIH: Brown University Summer Assistantship program and from the Brown Emerging Infectious Disease Scholars (EIDS) (5R25AI140490) **Dimitrios Farmakiotis, M.D.**, Astellas: Grant/Research Support|Merck: Grant/Research Support|Viracor: Advisor/Consultant|Viracor: Grant/Research Support

